# Therapeutic Effect and Location of GFP-Labeled Placental Mesenchymal Stem Cells on Hepatic Fibrosis in Rats

**DOI:** 10.1155/2017/1798260

**Published:** 2017-04-12

**Authors:** Jiong Yu, Guangshu Hao, Dan Wang, Jingqi Liu, Xiaotian Dong, Yanni Sun, Qiaoling Pan, Yang Li, Xiaowei Shi, Lanjuan Li, Hongcui Cao

**Affiliations:** ^1^State Key Laboratory for the Diagnosis and Treatment of Infectious Diseases, First Affiliated Hospital, College of Medicine, Zhejiang University and Collaborative Innovation Center for Diagnosis and Treatment of Infectious Diseases, 79 Qingchun Road, Hangzhou 310003, China; ^2^Gansu Provincial Maternity and Child-Care Hospital, 143 Qilihe North Street, Lanzhou 730050, China; ^3^Obstetrical Department, First Affiliated Hospital, College of Medicine, Zhejiang University, 79 Qingchun Road, Hangzhou 310003, China; ^4^Chu Kochen Honors College, Zhejiang University, 866 Yuhangtang Road, Hangzhou 310058, China

## Abstract

*Background*. Liver fibrosis is a chronic progressive liver disease, but no established effective treatment exists except for liver transplantation. The present study was designed to investigate the effect of human placenta mesenchymal stem cells (hPMSCs) expressing green fluorescent protein (GFP) on carbon tetrachloride- (CCl_4_-) induced liver fibrosis in rats. *Methods*. Liver fibrosis was induced by subcutaneous injection with CCl_4_; hPMSCs were directly transplanted into rats through the caudal vein. The therapeutic efficacy of hPMSCs on liver fibrosis was measured by liver function tests, liver elastography, histopathology, Masson's trichrome and Sirius red staining, and immunohistochemical studies. The expression levels of fibrotic markers, transforming growth factor *β*1 (TGF-*β*1) and *α*-smooth muscle actin (*α*-SMA), were assessed using real-time polymerase chain reaction. *Results*. We demonstrated that liver fibrosis was significantly dampened in the hPMSC transplantation group according to the Laennec fibrosis scoring system and histological data. The Sirius red-stained collagen area and the elastography score were significantly reduced in the hPMSC-treated group. Meanwhile, hPMSC administration significantly decreased TGF-*β*1 and *α*-SMA expression and enhanced liver functions in CCl_4_-induced fibrotic rats. *Conclusion*. This study indicates that transplantation of hPMSCs could repair liver fibrosis induced by CCl_4_ in rats, which may serve as a valuable therapeutic approach to treat liver diseases.

## 1. Introduction

Liver fibrosis is a common chronic progressive liver disease caused by one or more etiologies such as viruses, alcohol, parasites, autoimmune reactions, long-term drug damage, or repeated effects of the formation of diffuse liver damage [1]. At home and abroad, orthotopic liver transplantation is currently recognized as the most effective treatment, but a shortage of donor organs and other issues limit the wide application of the treatment [2, 3]. Therefore, it is extremely important to find other effective methods to treat liver fibrosis.

With the development of cell transplantation in recent years, mesenchymal stem cells (MSCs) have attracted more and more attention in the treatment of liver fibrosis [4, 5]. MSCs are a class of mesoderm-derived adult stem cells with a high self-renewal capacity and multidirectional differentiation potential. In addition, MSCs also have low immunogenicity, immunomodulatory, and anti-inflammatory effects [6, 7].

MSCs are widely found in the bone marrow, adipose tissue, placenta, and cord blood as well as other tissues and organs [8–10]. Among them, bone marrow-derived MSC (BMSC) is currently the most widely studied MSC, but limited by its potential damages to the donors, its low quantities available, and the existence of ethical problems. Human placenta-derived mesenchymal stem cell (hPMSC), a promising source of MSCs, has biological characteristics similar to those of BMSC and is readily available from a rich source through noninvasive methods, is free of ethical issues, and has a higher differentiation capacity and lower immunogenicity, as well as other advantages [11–14]. All of these advantages indicate the broad potential applications for hPMSCs.

In this study, we used hPMSCs with green fluorescent protein (GFP^+^ hPMSCs) and the corresponding imaging modality to provide a novel approach to continuously track and quantify the fate of hPMSCs in vivo, investigated the effect of GFP^+^ hPMSCs on hepatic fibrosis in a carbon tetrachloride- (CCl_4_-) induced fibrotic rat model [15], and provided an experimental and theoretical basis for the clinical use of hPMSC transplantation for the treatment of liver diseases.

## 2. Materials and Methods

### 2.1. Cell Culture and Gene Transduction

hPMSCs were obtained and transduced as previously described [12, 16]. Briefly, hPMSCs were cultured in special medium (Mesen Cult® Human Basal Medium plus MesenCult® Human Supplement, STEMCELL Technologies Inc., Vancouver, Canada) with a standard humidified incubator (HERAcell®150, Thermo Fisher Scientific Inc.) with 5% CO_2_ at 37°C. When the hPMSCs were stabilized and reached 50% confluence, the recombinant lentivirus (Gene Pharma, Shanghai, China) was added to the medium to perform the transfection experiment as previously described. Then, the culture medium was replaced with fresh medium mixed with 10 *μ*g puromycin (Sigma-Aldrich Co. LLC, St. Louis, MO, USA); the cells were continuously cultured and trypsinized using 0.25% (*w*/*v*) trypsin/ethylenediaminetetraacetic acid (EDTA, Invitrogen, Carlsbad, CA, USA) and passaged at a 1 : 3 dilution upon reaching 70–80% confluence. Finally, the verification of GFP successful expression was done following the previous experiment. All protocols for human tissue and cell handling were approved by the Research Ethics Committee of First Affiliated Hospital, School of Medicine, Zhejiang University (Reference number 2013-272).

### 2.2. Liver Fibrosis Animal Model

Six-week-old, specific-pathogen-free, male Sprague Dawley rats weighing 180 g to 200 g were obtained from the Experimental Animal Center of Zhejiang Academy of Medical Sciences and housed in an air-conditioned animal room with 50% humidity and a 12 h daylight/darkness cycle. All rats were treated according to protocols approved by the Research Ethics Committee of the First Affiliated Hospital, School of Medicine, Zhejiang University. To induce liver fibrosis, rats were given a subcutaneous injection of 50% CCl_4_ (Wuxi Zhanwang Chemical Co. Ltd., Wuxi, China) mixed with olive oil (Sangon Biotech Co. Ltd., Shanghai, China) at a dose of 0.5 mL per 100 g of body weight twice per week for 8 weeks. After 8 weeks, five model rats were selected randomly to verify liver fibrosis by pathological testing of liver tissue.

#### 2.2.1. Experimental Groups and Cell Transplantation

The animals were randomly divided into three groups as follows: group I (saline control group, *n* = 24), which received saline instead of carbon tetrachloride injection and cell transplantation (passages 3 to 6; 2.0 × 10^6^ hPMSCs in 1 mL saline) by caudal vein injection; group II (hPMSC-treated group, *n* = 24), fibrosis models with hPMSC transplantation via the caudal vein (passages 3 to 6; 2.0 × 10^6^ hPMSCs in 1 mL saline); and group III (untreated fibrosis group, *n* = 24), which received saline by caudal vein injection instead of cell transplantation. Fluorescence imaging and liver shear-wave elastography were performed on the rats, and the rats were then killed at 1 week (1 w), 4 weeks (4 w), 8 weeks (8 w), and 12 weeks (12 w) post transplantation, after which liver tissue and serum were extracted for follow-up analyses.

#### 2.2.2. Biochemical Analysis and Serum Determination

Blood samples were acquired from rats at each time point, placed at room temperature for 30 minutes, and then centrifuged for 15 minutes at 3000 rpm (Sorvall® Biofuge Stratos, Thermo, Germany), and the serum was collected. Then, albumin (ALB), alanine aminotransferase (ALT), aspartate aminotransferase (AST), alkaline phosphatase (ALP), and total bilirubin (TBIL), direct bilirubin (DBIL), gamma-glutamyl transpeptidase (*γ*-GT) concentrations were assessed using an automated biochemical analyzer (Abbott Aeroset, Abbott Laboratories, Chicago, IL, USA). The levels of hepatic fibrogenesis indicators, such as laminin (LN) and hyaluronan (HA), were measured using commercial enzyme-linked immunosorbent assay (ELISA) kits (R&D Systems Inc., Minneapolis, USA and Abnova Corporation, Taiwan) according to the manufacturer's instructions.

#### 2.2.3. Liver Evaluation with Elastography and Histology

First, we performed liver shear-wave elastography (Siemens AG, Berlin, Germany) to assess the degree of hepatic fibrosis in rats at various time points. In the assessment of chronic liver damage, hepatic elastography is the most reproducible. In the scoring system, liver fibrosis is evaluated semiquantitatively [17, 18]. As described below, the rats were weighed and anesthetized by subcutaneous injection of chloral hydrate (Sangon Biotech Co. Ltd., Shanghai, China) at a dose of 0.3 mL/100 g, and then the liver shear-wave elastography of rats was examined after rat thoracic hair was removed with a shaver.

To further verify the extent of the rat liver fibrosis, the hepatic specimens were fixed in 4% paraformaldehyde and embedded in paraffin, then deparaffinized and rehydrated with distilled water, and stained with hematoxylin and eosin (H&E), Masson's trichrome (MTC) and Sirius red using a Masson's trichrome staining kit (Sigma-Aldrich Co. LLC, MO, USA) and Sirius red staining kit (Wuhan Goodbio Technology Co. Ltd., Wuhan, China) according to the manufacturer's instructions. First, the Laennec fibrosis scoring system was used for quantitative analysis of fibrosis [19]. Additionally, Sirius red staining was performed to estimate the amount of collagen in the liver tissue; the collagen content was quantified as a percentage of the total area positive for Sirius red stain using ImageJ version 1.35s (National Institutes of Health, Bethesda, MD).

#### 2.2.4. Liver Immunohistochemistry

The paraffin sections were deparaffinized and rehydrated. To inactivate the endogenous peroxidase, the sections were incubated in 3% hydrogen peroxide-methanol solution for 10 minutes. Then, antigen retrieval was performed by microwave (Midea Group Co. Ltd., Guangdong, China) treatment in 0.01 M citrate salt buffer (pH 6.0, Wuhan Goodbio Technology Co. Ltd., Wuhan, China) for 15 minutes and blocked in 5% BSA for 45 minutes at room temperature. Sections were incubated overnight at 4°C with the diluted primary antibody against human ALB (1 : 250; Abcam, UK), alpha-fetoprotein (AFP, 1 : 100; Abcam, UK), cytokeratin 18 (CK18, 1 : 100; Abcam, UK) as well as antirat *α*-smooth muscle actin (*α*-SMA, 1 : 400; Abcam, UK) and TGF-*β*1 (1 : 500; Abcam, UK). The next day, the sections were washed 3 times with phosphate-buffered saline (PBS, pH 7.2 ± 0.1, GenomSciences, Hangzhou, China) and then incubated at 37°C in an incubator for 60 minutes with horseradish peroxidase-conjugated secondary antibodies (1 mg/mL, 1 : 1000; Abcam, UK). A brown color was developed with diaminobenzidine tetrahydrochloride solution (DAB kit, Abcam, UK) for 10 min, washed in distilled water 3 times, and counterstained with hematoxylin for 5 minutes at room temperature. Finally, the liver sections were sealed with neutral resin and examined microscopically.

#### 2.2.5. Reverse Transcription-Polymerase Chain Reaction and Quantitative Real-Time Polymerase Chain Reaction

Total RNA from each sample was extracted with the Trizol reagent (Invitrogen, USA). The RNA purity and quantity were measured by determining absorbance at 260 and 280 nm, and 1 *μ*g of RNA was used as the template for cDNA synthesis using the QuantiTect Reverse Transcription Kit (TAKARA Biotechnology (Dalian) Co. Ltd., Dalian, China) according to the manufacturer's instructions. The primers for the target products were designed as shown in Supplementary Table S1 available online at https://doi.org/10.1155/2017/1798260. Polymerase chain reaction (PCR) amplification was subsequently carried out in a PCR thermal cycler (Life Technologies, Carlsbad, California, USA). The PCR products were separated by 1.2% agarose gel electrophoresis containing gelred. Then, the images were acquired using a gel documentation system (Syngene GBox-HR Gel Doc System, UK). The quantitative real-time polymerase chain reaction (qRT-PCR) was performed to measure the mRNA levels of TGF-*β*1 and *α*-SMA (QuantiTect SYBR Green RT-PCR kit, TAKARA Biotechnology (Dalian) Co. Ltd.) with a 7500 Real Time System (Life Technologies, Carlsbad, California, USA). All PCR products were normalized to the expression levels of *β*-actin used as an internal standard.

### 2.3. Statistical Analysis

Statistical analysis was performed using the SPSS 19.0 software (SPSS Inc., Chicago, IL, USA). All data were presented as the mean ± standard deviation (SD). Statistical comparison among three groups was performed using a one-way ANOVA analysis, and a comparison between the groups was evaluated using Student's *t*-test. *p* < 0.05 was considered statistically significant.

## 3. Results

### 3.1. Construction of hPMSCs Transfected with Green Fluorescent Protein

hPMSCs that were cultured in normal conditions showed a fibroblast-like morphology (see Supplementary Figure S1A). Then, hPMSCs were transduced with a lentiviral vector encoding GFP at a multiplicity of infection (MOI) of 100 : 1. The expression of GFP visualized by fluorescence/phase-contrast microscopy was used to evaluate the successful lentiviral transduction in cultured hPMSCs (see Supplementary Figure S1B).

### 3.2. Enhanced Resolution of Liver Fibrosis by Transplantation of hPMSCs

**Figure 1:**
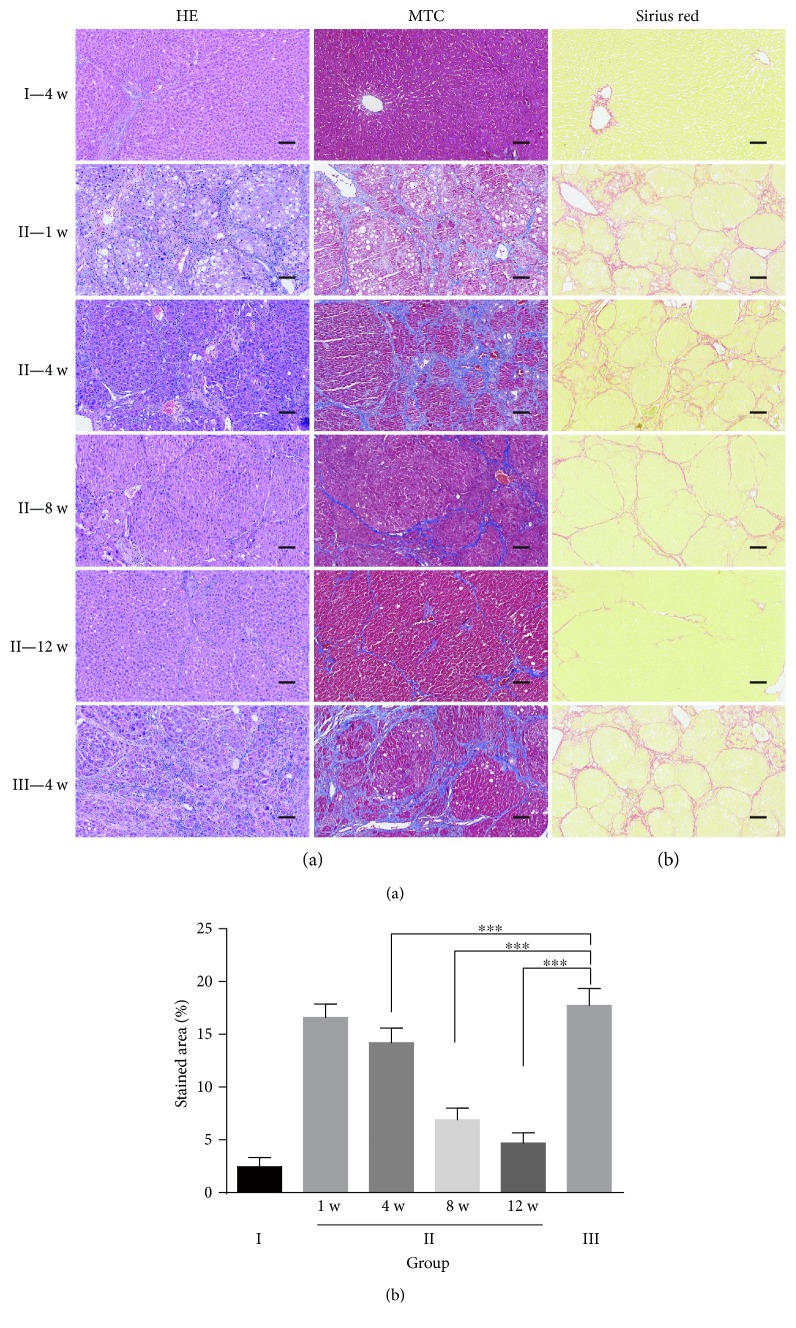
Assessment of liver fibrosis in the different experimental groups by tissue dyes. (a) Histological analysis was evaluated by H&E and MTC staining; (b-c) representative micrograph of hepatic tissue stained with Sirius red (b), and the relative expression of collagen was quantified using ImageJ analysis (c), *n* = 6.

The results from the histologic liver evaluation showed that transplantation of hPMSCs (group II) had a more beneficial effect on the recovery of liver fibrosis than saline treatment in the untreated fibrosis group (group III), exhibited as strong H&E and MTC staining, showing regenerative nodules and progressive reduction in the amount of collagen occurred in the fibrous septa, especially after the eighth week of transplantation. The degree of fibrosis was significantly improved by hPMSC transplantation. In group II rats on twelve weeks after cell transplantation, the liver was close to the normal structure of the liver, and the architecture of the impaired hepatic lobule returned to normal, as it was no different from the liver structure of rats in the saline control group (group I) (Figure 1(a)). Consistently, the results from the Laennec fibrosis scoring system showed that group II had a relatively lower average score than group III (Table 1). The Sirius red staining results revealed that the collagen-stained area in liver sections was more decreased in the hPMSC-treated group than that in the untreated fibrosis group, and the collagen-stained area decreased with time in the hPMSC-treated group. However, the collagen-stained area maintained a high level in the untreated fibrosis group. Almost no collagen deposition was observed in group I (Figures 1(b) and 1(c)).

**Figure 2:**
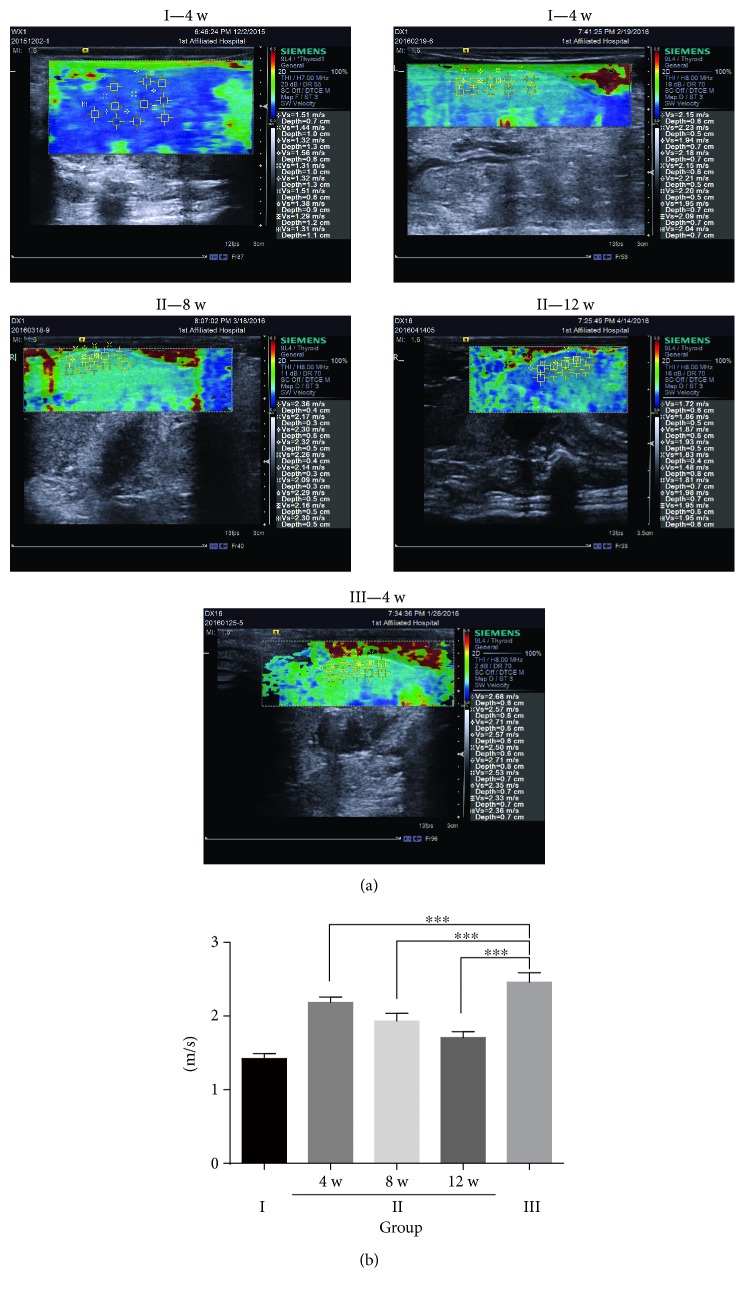
Semiquantitative assessment of liver fibrosis in the different experimental groups by shear-wave elastography. (a) Representative micrograph of liver elastography in the three groups. Saline control group rats (group I), untreated fibrosis animals (group III), fibrotic animals transplanted with hPMSCs (group II) at four weeks, eight weeks, and twelve weeks after transplantation (4 w, 8 w, and 12 w). Images are representative images from *n* = 3 rats at each time point. The data are presented as the mean ± SD (error bars) and were statistically analyzed using one-way ANOVA.^∗∗∗^*p* < 0.001.

The liver elastography showed a similar result. In the untreated fibrosis group, the stiffness score was significantly upregulated, but it was significantly decreased in the hPMSC-treated group (Figure 2). All of these data clearly suggest that hPMSCs were an effective therapeutic method for rat hepatic fibrosis and attenuated the extent of hepatic fibrosis in experimental rats.

### 3.3. Biochemical Analysis

**Figure 3:**
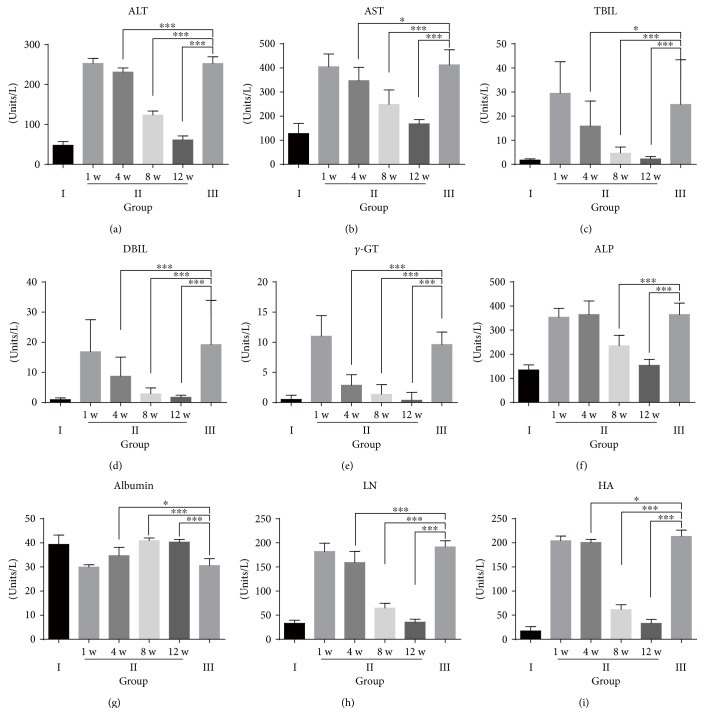
Biochemical analyses. (a) Alanine aminotransferase (ALT); (b) aspartate aminotransferase (AST); (c) total bilirubin (TBIL); (d) direct bilirubin (DBIL); (e) gamma glutamyl transpeptidase (*γ*-GT); (f) alkaline phosphatase (ALP); (g) albumin (ALB); (h) laminin (LN); (i) hyaluronic acid (HA). Saline control group rats (group I), untreated fibrosis animals (group III), and fibrotic animals transplanted with hPMSCs (group II) and sacrificed one week, four weeks, eight weeks, or twelve weeks after transplantation (1 w, 4 w, 8 w, or 12 w). (a–g) Group I, *n* = 20; group II, *n* = 8; and group III, *n* = 8. (h–i) Group I, *n* = 9; group II, *n* = 8; and group III, *n* = 8. Data represent the means ± SD and were statistically analyzed by one-way ANOVA. ^∗^*p* < 0.05, ^∗∗^*p* < 0.01, and ^∗∗∗^*p* < 0.001.

Biochemical analyses were performed to assess the restoration of the liver functions and hepatic fibrosis. The results showed that ALT, AST, TBIL, DBIL, and *γ*-GT levels were significantly decreased in the hPMSC-treated group (Figures 3(a)–3(e)) and approached normal levels 12 weeks after cell transplantation. The levels of ALP showed a similar pattern (Figure 3(f)). In addition, the serum level of albumin in the hPMSC-treated group was higher than that in the untreated fibrosis group and approached the normal level of the saline control group at 12 weeks after transplantation (Figure 3(g)).

The serum levels of LN and HA in the hPMSC-treated group were lower than those in the untreated fibrosis group. The level of LN approached the level in the saline control group at the last time point examined, but the level of HA was still higher than the normal level at that time (Figures 3(h) and 3(i)).

### 3.4. Localization and Fate of the Transplanted hPMSCs in the Liver

**Figure 4:**
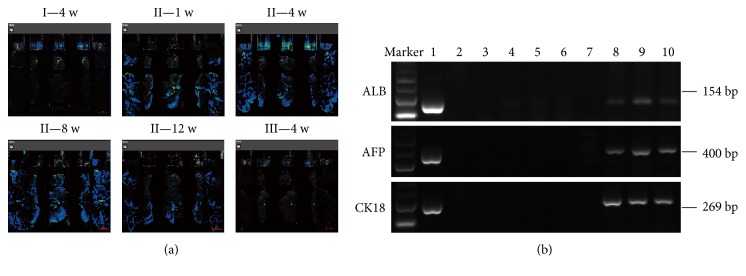
Localization and fate of the transplanted hPMSCs in rat livers of different experimental groups. (a) Representative figures of liver fluorescence are from *n* = 6 rats from each group at each time point; (b) RT-PCR of human-specific markers. Lane 1, positive control (human liver for ALB and CK18, human liver cancer for AFP); lane 2, the liver of group III as a negative control; lane 3 to 6, hPMSC transplantation at 1 w, 4 w, 8 w, and 12 w in group I. Human AFP and CK18 were not detected at the four time points, but ALB was detected in lane 4. Lane 7 to 10, hPMSC transplantation at 1 w, 4 w, 8 w, and 12 w in group II. Human ALB, AFP, and CK18 were all detected at the three later time points but not at 1 w.

To detect the colonization and differentiation of hPMSCs in the rat after transplantation, we used fluorescence microscopy to image the liver of the rats at the corresponding time points. As shown in Figure 4(a), in group II, fluorescence was detected in the liver tissue at all four time points after hPMSC transplantation, but the fluorescence signal was very weak at 12 weeks. However, in groups I and III, no fluorescence signal was detected in the liver. The liver tissue samples were photographed and fixed paraffin sections, and the expression of liver-specific markers AFP, ALB, and CK18 were detected by immunohistochemistry.

AFP, ALB, and CK18 mRNA were all detected in the liver tissue at 4 weeks, 8 weeks, and 12 weeks after transplantation through RT-PCR analysis using human-specific primers. Human AFP, ALB, and CK18 mRNA were not detected in the livers at 1 week after hPMSC transplantation, which was also confirmed by immunohistochemistry. No expression of human AFP, ALB, or CK18 was detected in group I or group III liver samples, except that human ALB mRNA expression was detected in one rat from group I (Figure 4(b)).

**Figure 5:**
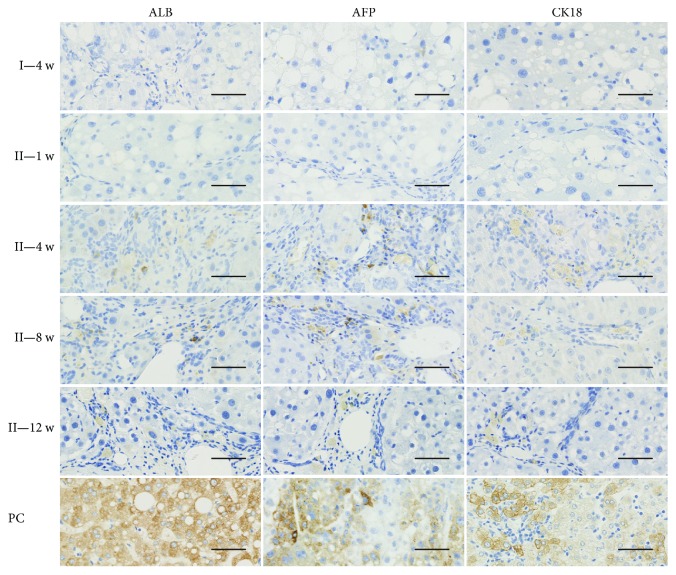
Hepatogenic differentiation of transplanted hPMSCs in rat livers (20x). Immunohistochemistry staining for human hepatic markers ALB, AFP, and CK18, light-brown coloration in cells means the positive staining results. Group III as a negative control; PC: human liver as positive control for ALB and CK18 and human liver cancer as a positive control for AFP; group II: fibrotic animals transplanted with hPMSCs and sacrificed one week, four weeks, eight weeks, or twelve weeks after transplantation (1 w, 4 w, 8 w, or 12 w). Scale bars: 100 *μ*m.

We obtained similar results through immunohistochemistry. As shown in Figure 5, in group II, positive staining for AFP, ALB, and CK18 was not observed in any liver specimen at 1 week after hPMSC transplantation. However, the expression of AFP-, ALB-, and CK18-positive cells was detected in all liver tissue of rats at 4 weeks after transplantation, and the positive cells were mainly scattered along the perivascular and the fibrous septa around the portal area. In addition, AFP-, ALB-, and CK18-positive cells were also detected at 8 and 12 weeks after hPMSC transplantation, but the number of positive cells decreased with time. In group III liver samples, no human AFP-, ALB-, or CK18-positive staining was detected (data not shown).

### 3.5. Inhibition of Hepatic Stellate Cell Activation by hPMSCs

**Figure 6:**
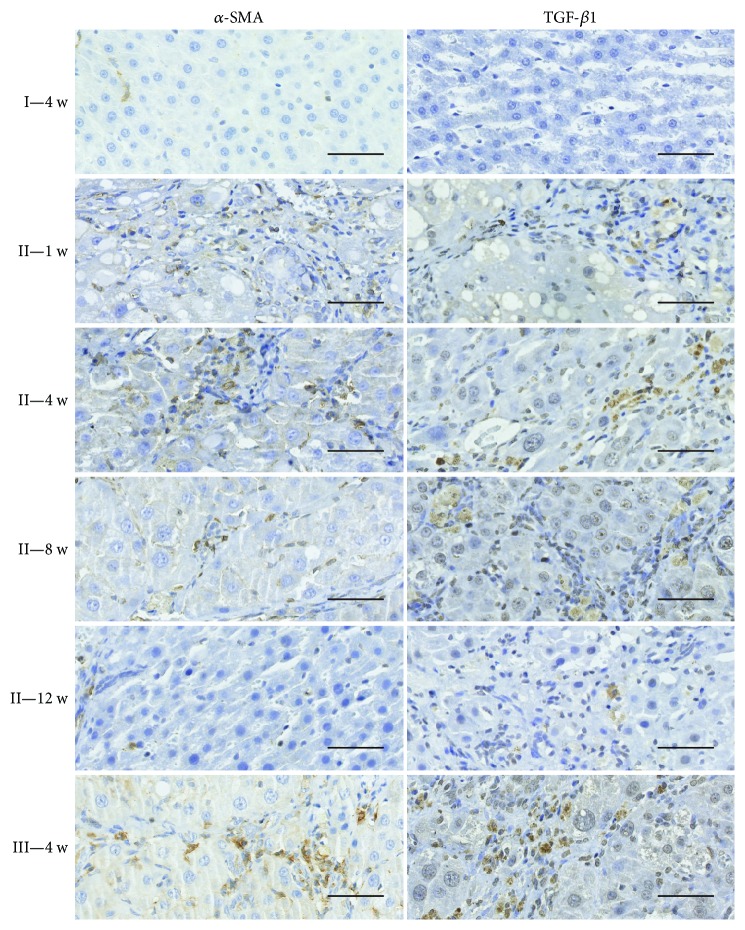
Immunohistochemistry results of *α*-SMA and TGF-*β*1 in the different experimental groups. Light-brown coloration in cells means the positive staining results. Immunohistochemical staining for *α*-SMA and TGF-*β*1 (20x), with a young rat liver as a negative control (NC), fibrotic animals (group II), fibrotic animals transplanted with hPMSCs (group II) sacrificed at one week, four weeks, eight weeks, or twelve weeks after transplantation (1 w, 4 w, 8 w, or 12 w). Scale bars: 100 *μ*m.

**Figure 7:**
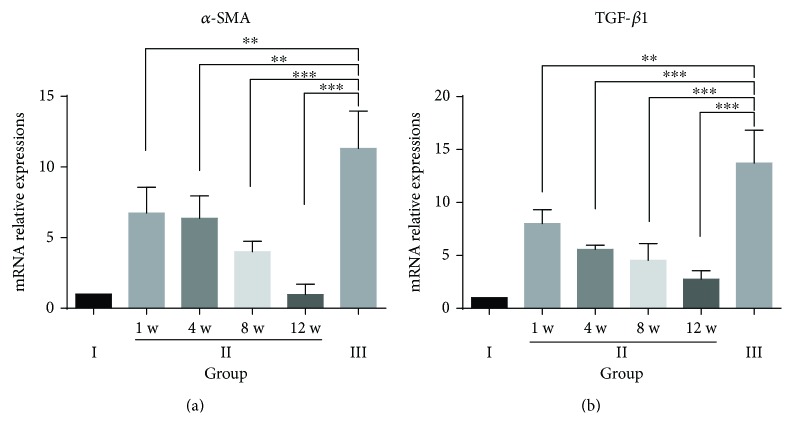
Relative mRNA expression levels of *α*-SMA and TGF-*β*1 in the different experimental groups. Quantitative real-time polymerase chain reaction (qRT-PCR) results from *n* = 6 rats from each group at each time point. The tests were performed in triplicate. The data are presented as the mean ± SD (error bars) and were statistically analyzed using Student's *t*-test. ^∗∗^*p* < 0.01, ^∗∗∗^*p* < 0.001.

It is well known that activation of hepatic stellate cells (HSCs) is currently recognized as a central event in the development of hepatic fibrosis, in which activated HSCs participate in the formation of hepatic fibrosis through proliferation and secretion of *α*-SMA protein, which is a marker of activated HSCs and plays a central role in extracellular matrix (ECM) production in response to liver damage. In addition, TGF-*β*1 is a fibrogenic master cytokine that plays a central role in the progression of ECM degradation. The results through immunohistochemistry (Figure 6) and qRT-PCR (Figure 7) revealed that *α*-SMA and TGF-*β*1 levels reached the highest value in the untreated fibrosis group (group III). However, *α*-SMA and TGF-*β*1 expression was significantly lower in the hPMSC-treated group (group II) than that in the untreated fibrosis group (group III). In addition, in group II, the levels of *α*-SMA and TGF-*β*1 decreased with time and approached normal levels (saline control group) 12 weeks after cell transplantation. These results indicated that hPMSC transplantation may prevent HSC differentiation and inhibit HSC activity.

## 4. Discussion

Recently, MSCs have been reported to potentially secrete organotrophic factors that protect cells from damage or activate endogenous restorative mechanisms to enhance fibrous matrix degradation and restore the injured liver [20, 21]. In this study, we investigated the therapeutic effect of hPMSCs in a CCl_4_-induced rat liver fibrosis model. We found that hPMSC transplantation exhibited an enhanced therapeutic effect compared to that observed without hPMSC transplantation. We performed fluorescence imaging of the rat liver at multiple time points to determine whether GFP-labeled hPMSCs were expressed in the rat liver. As an important means of tracking cells, GFP tagging has a strong advantage. Using fluorescent imaging devices, it was fairly easy to see whether GFP-labeled hPMSCs colonized in the liver as well as analyze the number and distribution of cells in the rat liver. These results, combined with the data from RT-PCR and immunohistochemistry, were much more credible. As the images showed, the expression of the fluorescent signal was detected at all four time points in the hPMSC-treated group (group II), but the signal intensity was weakest at 12 weeks. Nevertheless, our data clearly demonstrated the presence of transplanted hPMSCs in the damaged rat liver.

Cirrhosis and advanced fibrosis are generally considered irreversible conditions and are not diagnosed until the condition has reached an advanced stage [22]. Therefore, early diagnosis of liver fibrosis has become very important. In general, liver fibrosis and its severity are determined through H&E, MTC, and Sirius red and *α*-SMA staining of histological sections. In addition, we used B-ultrasonography, which has the advantages of less trauma, providing intuitive results and having less of an effect on the experimental animals and biological events that need to be observed to evaluate liver fibrosis in rats [23, 24]. In this study, shear-wave elastography of the liver was also a supplement to the results of the pathological analysis, making the experimental results more credible. Some studies have used the Metavir scoring system to classify liver fibrosis. However, due to a lack of accurate subclassification of cirrhosis using the Metavir scoring system, liver pathologists will often come to the wrong conclusions when assessing the therapeutic effect. Therefore, further better histological subclassification of cirrhosis is required. In this study, we applied the new Laennec system; these histologic criteria have been better defined to provide a more exact and detailed classification of cirrhotic stage [19, 25].

HSC activation and increased ECM synthesis paired with insufficient degradation play a central role in the change induced by hepatic fibrosis. The expression of *α*-SMA in the liver is an indicator for the HSC activation, indicating that activated HSCs expressing *α*-SMA are involved in the development and progression of hepatic fibrosis [26, 27]. In the present study, levels of *α*-SMA in the rat liver significantly decreased after hPMSC transplantation. This result may explain the reduced fibrogenesis in the hPMSC-transplanted liver.

The balance between ECM deposition and degradation is regulated by a variety of cytokines, the most important of which is TGF-*β*1 [28, 29]. TGF-*β*1 can inhibit the proliferation of hepatocytes, induce hepatocyte apoptosis, activate HSCs, promote the production of extracellular matrix, and inhibit the degradation of extracellular matrix. Furthermore, TGF-*β*1 can inhibit the production of collagenase and protease, promote tissue inhibitors such as tissue inhibitor of metalloproteinase production, and reduce ECM degradation [30, 31]. TGF-*β*1 can also indirectly affect ECM synthesis by acting on other cytokines [32]. As shown in this study, hPMSC transplantation into the rat liver reduced the level of TGF-*β*1, resulting in a decrease in the activation of hepatic stellate cells and reduction in hepatic fibrosis.

In conclusion, our study showed that MSCs from the human placenta could effectively cure liver fibrosis, reduce the activation of hepatic stellate cells, and restore liver functions. However, the underlying mechanism remains to be further clarified. Therefore, hPMSC therapy may be a new and effective strategy for the treatment of fibrotic liver disease in the future.

## Supplementary Material

Table S1. Primer sequences and conditions for RT-PCR or qRT-PCR. Figure S1. Construction of GFP-transfected hPMSCs. A: Micrographs of cultured hPMSCs at passage 3 (10×); B: hPMSCs with lentiviral transduction after 3 days.

## Figures and Tables

**Table 1:** Histological stage of hepatic fibrosis.

Group	Stage 0	Stage 1	Stage 2	Stage 3	Stage 4A	Stage 4B	Stage 4C	Average
Score	0	1	2	3	4	5	6	
Group I	24							0
Group II			1	5	8	10		4.125
Group III						9	15	5.625

## Abbreviations

AFP: Alpha-fetoprotein
ALB: Albumin
ALP: Alkaline phosphatase
ALT: Alanine aminotransferase
AST: Aspartate aminotransferase
CCl_4_: Carbon tetrachloride
CK18: Cytokeratin 18
DBIL: Direct bilirubin
ECM: Extracellular matrix
EDTA: Ethylenediaminetetraacetic acid
ELISA: Enzyme-linked immunosorbent assay
GFP: Green fluorescent protein
H&E: Hematoxylin and eosin
HA: Hyaluronan
hPMSCs: Human placental mesenchymal stem cells
HSC: Hepatic stellate cell
LN: Laminin
MOI: Multiplicity of infection
MSCs: Mesenchymal stem cells
MTC: Masson's trichrome
PBS: Phosphate-buffered saline
PCR: Polymerase chain reaction
RT-PCR: Reverse transcription-polymerase chain reaction
TBIL: Total bilirubin
TGF-*β*1: Transforming growth factor *β*1
*α*-SMA: *α*-Smooth muscle actin
*γ*-GT: Gamma-glutamyl transpeptidase.

## Additional Points

*Highlights*. The following are the highlights of the paper:

- Green fluorescent protein gene-transduced human placenta-derived mesenchymal stem cells (hPMSCs) and the corresponding imaging modality were used to provide a novel approach to continuously track and quantify the fate of hPMSCs in vivo and to investigate the effect of their transplantation on the carbon tetrachloride- (CCl_4_-) induced liver fibrosis in rats.
- Biochemical analyses, histology, immunohistochemistry, and liver shear-wave elastography were used to evaluate the improvement on hepatic fibrosis after hPMSC transplantation.
- hPMSCs had an enhanced therapeutic effect for hepatic fibrosis compared to non-hPMSCs possibly via their differentiation and inhibition of hepatic stellate cell activation.
