# Supplementary material for: Therapeutic Effect and Location of GFP-Labeled Placental Mesenchymal Stem Cells on Hepatic Fibrosis in Rats
Source: Stem Cells Int. 2017 Apr 12;2017:1798260. doi: 10.1155/2017/1798260 (PMC5405597; doi:10.1155/2017/1798260)
Supplement: Supplementary file 1 — Table S1. Primer sequences and conditions for RT-PCR or qRT-PCR. Figure S1. Construction of GFP-transfected hPMSCs. A: Micrographs of cultured hPMSCs at passage 3 (10×); B: hPMSCs with lentiviral transduction after 3 days. [file 1798260.f1.doc]

**Supplemental data**

**Therapeutic effect and location of GFP labeled placental mesenchymal stem cells on hepatic fibrosis in rats**

Jiong Yu^1*^, Guangshu Hao^2*^, Dan Wang^1^, Jingqi Liu^1^, Xiaotian Dong^1^, Yanni Sun^1^, Qiaoling Pan^1^, Xiaowei Shi^3^, Jinfeng Yang^1^, Yang Li^2^, Lanjuan Li^1^, Hongcui Cao^1^**^†^**

1 State Key Laboratory for the Diagnosis and Treatment of Infectious Diseases, First Affiliated Hospital, College of Medicine, Zhejiang University; Collaborative Innovation Center for Diagnosis and Treatment of Infectious Diseases, 79 Qingchun Rd., Hangzhou City 310003, China

2 Gansu Provincial Maternity and Child-care Hospital, 143 Qilihe North Street, Lanzhou City 730050, China

3 [Obstetrical](javascript:void(0);) [department](javascript:void(0);), First Affiliated Hospital, College of Medicine, Zhejiang University; Collaborative Innovation Center for Diagnosis and Treatment of Infectious Diseases, 79 Qingchun Rd., Hangzhou City 310003, China

4 Chu Kochen Honors College, Zhejiang University, 866 Yuhangtang Rd., Hangzhou City 310058, China

* These authors contributed equally to this work.

**†Correspondence author:**

Hongcui Cao, State Key Laboratory for Diagnosis and Treatment of Infectious Diseases, First Affiliated Hospital, College of Medicine, Zhejiang University; Collaborative Innovation Center for Diagnosis and Treatment of Infectious Diseases,79 Qingchun Rd., Hangzhou City 310003, China.Tel: 86-571-87236451; Fax: 86-571-87236459

E-mail: [hccao@zju.edu.cn](mailto:hccao@zju.edu.cn)

Table S1. Primer sequences and conditions for RT-PCR or qRT-PCR

| Primer | | | **Sequence** | **Cycle** | |
| --- | --- | --- | --- | --- | --- |
| ALB | 5’- CACAGTTGCAACTCTTCGTGAAAC-3’  5’- AGCAGTGCACATCACATCAACC-3‘ | | | | 94°C/30 s; 60°C/40 s; 72°C/60 s |
| CK18 | | 5’- CACCGTCGTCCGCAAAGCCT-3’  5’- CCTGCCAGACCCCCGGCTAT-3’ | | | 94°C/30 s; 62°C/40 s; 72°C/60 s |
| AFP | | 5’- GCATGTGCAGTAATGAAAAAT-3’  5’- GAACAAAACTTGCCAAGAAGAT-3’ | | | 94°C/30 s; 58°C/40 s; 72°C/60 s |
| β-actin | | 5’-GAGCGGGAAATCGTGCGTGACATT-3’  5’-GATGGAGTTGAAGGTAGTTTCGTG-3’ | | | 94°C/30 s; 58°C/40 s; 72°C/60 s |
| TGF-β1 | | 5’- CCTGGAAAGGGCTCAACAC -3’  5’- CAGTTCTTCTCTGTGGAGCTGA -3’ | | | 95°C/30 s; 95°C/5s; 60°C/34 s |
| α-SMA | | 5‘- CGATAGAACACGGCATCATCAC -3’  5’- GCATAGCCCTCATAGATAGGCA -3’ | | | 95°C/30 s; 95°C/5s; 60°C/34 s |
| β-actin | 5’-GAGCGGGAAATCGTGCGTGACATT-3’  5’-GATGGAGTTGAAGGTAGTTTCGTG-3’ | | | | 95°C/30 s; 95°C/5s; 60°C/34 s |

Figure S1.


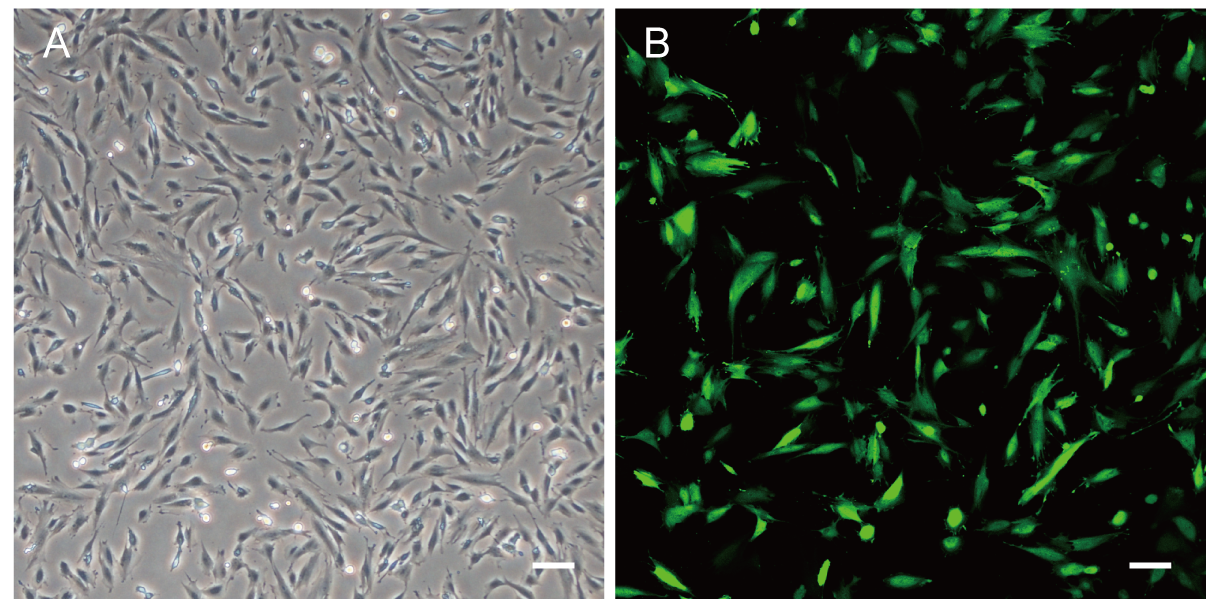


Construction of GFP-transfected hPMSCs.

A: Micrographs of cultured hPMSCs at passage 3 (10×); B: hPMSCs with lentiviral transduction after 3 days.
